# Epoxy-Based Shape-Memory Actuators Obtained via Dual-Curing of Off-Stoichiometric “Thiol–Epoxy” Mixtures

**DOI:** 10.3390/polym9030113

**Published:** 2017-03-21

**Authors:** Alberto Belmonte, Claudio Russo, Veronica Ambrogi, Xavier Fernández-Francos, Silvia De la Flor

**Affiliations:** 1 Department of Mechanical Engineering, Universitat Rovira i Virgili, Av. Països Catalans 26, 43007 Tarragona, Spain; albertofrancisco.belmonte@urv.cat (A.B.); claudio.russo6@studenti.unina.it (C.R.); 2 Department of Chemical, Materials and Production Engineering, University of Naples “Federico II”, Piazzale Tecchio, 80, 80125 Napoli, Italy; ambrogi@unina.it; 3 Institute for Polymers, Composites and Biomaterials, National Research Council of Italy, Via Campi Flegrei, 34, 80078 Pozzuoli, Italy; 4 Thermodynamics Laboratory, ETSEIB, Universitat Politècnica de Catalunya, Av. Diagonal 647, 08028 Barcelona, Spain; xavier.fernandez@mmt.upc.edu

**Keywords:** dual-curing, shape-memory polymer, actuator, thiol–epoxy, click chemistry, unconstrained, partially-constrained, fully-constrained

## Abstract

In this work, epoxy-based shape-memory actuators have been developed by taking advantage of the sequential dual-curing of off-stoichiometric “thiol–epoxy” systems. Bent-shaped designs for flexural actuation were obtained thanks to the easy processing of these materials in the intermediate stage (after the first curing process), and successfully fixed through the second curing process. The samples were programmed into a flat temporary-shape and the recovery-process was analyzed in unconstrained, partially-constrained and fully-constrained conditions using a dynamic mechanical analyzer (DMA). Different “thiol–epoxy” systems and off-stoichiometric ratios were used to analyze the effect of the network structure on the actuation performance. The results evidenced the possibility to take advantage of the flexural recovery as a potential actuator, the operation of which can be modulated by changing the network structure and properties of the material. Under unconstrained-recovery conditions, faster and narrower recovery-processes (an average speed up to 80%/min) are attained by using materials with homogeneous network structure, while in partially- or fully-constrained conditions, a higher crosslinking density and the presence of crosslinks of higher functionality lead to a higher amount of energy released during the recovery-process, thus, increasing the work or the force released. Finally, an easy approach for the prediction of the work released by the shape-memory actuator has been proposed.

## 1. Introduction

Shape-memory polymers (SMPs) are those materials capable of attaining and retaining a temporary-shape during a long period of time upon a so-called programming process. The programming process usually consists of a thermomechanical process in which the material is firstly heated up to a temperature close to or above a network structural transition (*T*_prog_), then mechanically deformed to produce a desired shape, and finally cooled down to a temperature below the network structural transition while keeping the applied deformation so that the programmed shape can be retained after removal of the mechanical load [1]. Therefore, the shape-memory capability of a polymer is not an intrinsic property of the polymer, but an external procedure is necessary. Nevertheless, it demands specific network structural properties. Soft and hard phases must coexist within the network, the hard phase responsible for memorizing the permanent shape and the soft phase allowing the shape-transformation [2,3]. One of the most usual SMPs are glassy, covalently or physically crosslinked polymers, therefore the crosslinks are the hard phase and the glass transition or melting process responsible for the shape-transformation is driven by the flexible chains or soft phase [4].

Over the last years, two main branches of research have been the focus of the scientific community: on one side, the enhancement and control of the shape-memory response (SMR) by adjusting network structural parameters, changing programming and recovery conditions and modeling the behavior [5,6,7,8,9]; on the other side, the development of new and challenging SMPs in order to fulfill some inherent issues, such as the need of programming after each cycle [10,11]. Nevertheless, one of the main issues lies in the development of SMPs with complex designs demanded in a wide range of industrial applications, such as structural, aerospace and nautical applications or in the development of bioinspired devices [12,13]. In such applications, the material is commonly required to work in aggressive environments; thus, high thermal and mechanical resistance are of utmost relevance. Epoxy-based thermosets are widely used in this field of applications due to the high thermal and mechanical resistance, great insulation properties and good chemical resistance [14]. Moreover, epoxy-based SMPs have shown excellent response, with high fixation and recovery ratios as well as rapid and tunable recovery-processes [15,16]. In this sense, they are excellent candidates to fulfill the operational requirements of such applications. However, the development of complex-shaped designs is a great challenge due to the hard control of the crosslinking during the curing process of epoxy resins making difficult further processing. One approach to overcome the curing limitations lies in the use of the so-called vitrimers [17,18], crosslinked polymers which, upon thermal treatment, can modify their chemical bonds to adopt a new permanent shape. Nevertheless, this process demands high consumption of energy for the network reconfiguration to take place and could lead to undesired final properties. Our research group has recently shown an effective approach to obtain complex-shaped epoxy-based materials through a two stage curing procedure. The sequential dual-curing of off-stoichiometric “thiol–epoxy” mixtures using tertiary amines as catalyst leads to the formation of an intermediate, stable, solid-like and conformable material after an initial and low energy consuming polymerization process (the “thiol–epoxy” polycondensation), that can be further processed into several complex shapes which are fixed through a second polymerization step triggered at higher temperature (the epoxy homopolymerization of the remaining epoxy groups) [19,20].

The great advantage on using such dual-curing methodology is the possibility of preparing materials with easy and efficient control of the final network structural properties, enhancing the thermomechanical properties of the common click “thiol–epoxy” thermosets. Through different “thiol” and “epoxy” compounds and varying the “thiol–epoxy” ratio of the off-stoichiometric mixture, a wide range of materials are produced: from low to highly crosslinked network structures, homogeneous and tailored glass transition processes and highly deformable glassy materials [19]. Moreover, these materials are potential candidates for shape-memory applications. On the one hand, it has been shown that the “thiol–epoxy” network leads to SMPs with enhanced response: high performance fixing and recovering the original shape, rapid recovery-process, high strength and extended deformation limits [21,22,23]. On the other hand, thanks to the possibility of forming complex-shaped designs, the final material is an interesting candidate for many of the mentioned applications.

To this end, in this study, we focus on the characterization of these materials as potential shape-memory polymers for actuation purposes. Shape-memory actuators are widely used in industrial applications as smart mechanisms for autonomous control. Taking into account that real operational scenarios usually involve flexural instead of tension or compression actuation designs, bent-shaped SMPs with different network structural properties have been processed from the intermediate material. The performance has been analyzed using the DMA equipment, which allows precise analysis and total control in flexural mode. In order to fully characterize the shape-memory response as an actuator, experiments in unconstrained, partially-constrained and fully-constrained conditions have been carried out, thus, from completely free-recovery conditions, passing through real situations in which the SMP behaves against an external force, to the fully impeded condition in which a recovery-force instead of shape is generated. Moreover, in order to enhance and evaluate the actuation performance, a highly functional epoxy resin, Epon^TM^ SU8, has been incorporated in a specific amount to the “thiol–epoxy” networks. It is known that the increase of the crosslinking density and the nature of the crosslinks have a crucial effect in the efficiency storing and releasing the energy associated to the shape-memory effect (SME) [5,24,25]. Taking into account the above, the incorporation of a large and spatially hindered molecule chemically attached to the network is expected to enhance the actuation performance.

## 2. Experimental Section

### 2.1. Materials and Methodology

Diglycidyl ether of bisphenol A (DGEBA, GY240, Huntsman, Everberg, Belgium) with an epoxy equivalent weight of 182 g/eq. was used as the main epoxy resin. EPON^TM^ resin SU8 with an epoxy equivalent average weight of 215 g/eq. was used as a highly functional network modifier (average functionality of 8 epoxy groups per molecule) in a molar proportion of 3:7 (SU8:DGEBA). Pentaerythritoltetrakis (3-mercaptopropionate) (S4) with a thiol equivalent weight of 122.17 g/eq. and trimethylolpropane tris(3-mercaptopropionate) (S3) with a thiol equivalent weight of 132.85 g/eq. (Sigma-Aldrich, St. Louis, MO, USA) were used as curing agents in an under-stoichiometric proportion with respect to the epoxy groups in the system. 1-methylimidazole (1MI) with a molecular weight of 82.1 g/mol (Sigma-Aldrich, St. Louis, MO, USA) was used as catalyst in a proportion of 1 phr (parts of catalyst per hundred parts of the whole mixture). The DGEBA was dried under vacuum overnight at 80 °C before use. The other reagents were used as received without further purification.

Different off-stoichiometric “thiol–epoxy” systems were chosen in order to study the SMR in relation with the network structural properties. The composition of the different formulations is shown in Table 1. According to our previous work [19], mixtures of DGEBA with both S3 and S4 thiol compounds separately, were chosen as the main systems of study. In addition, the SU8 resin was added in a specific proportion to the S3-DGEBA system in order to study the effect of incorporating a highly functional and large molecule in the network structure with the SMR. The SU8:DGEBA weight ratio was 3:7 to ensure a substantial increase of the *T*_g_ and crosslinking density while avoiding the formation of extremely brittle materials. The S4-DGEBA system was not considered due to the formation of extremely brittle materials when adding very small amounts of SU8 which do not satisfy the requirements for shape-memory purposes.

For each system, different “thiol–epoxy” ratios were chosen to study the effect of increasing the amount of epoxy excess with the SMR. Taking advantage of the characterization done in our previous work [19], the ratios were selected from the critical ratio (*r*_c_), which is the minimum “thiol–epoxy” ratio to form a gelled and therefore solid-like and conformable intermediate material after the 1st curing stage, up to a ratio in which both, the intermediate and final materials, have very similar properties and therefore the duality of the system is lost in terms of applicability. From the experimental data, it has been considered *r* = 0.9 the upper limit. In this way, all the formulations of study allow the processing of bent-shaped designs for the further shape-memory characterization as actuators. The *r*_c_ of the new system of study (S3-SU8-DGEBA) has been determined following the same experimental procedure as in our previous work [19]: intermediate materials cured from the theoretical *r*_c_ (calculated using the Flory equation) were heated up and the physical behavior observed. Non-gelled networks lead to undesired material flow and therefore shape-loosing. By increasing the “thiol–epoxy” ratio, the flow is reduced until no shape-loosing is appreciated. This ratio is then checked through rheological analysis to confirm that gelation is taking place and therefore it is stipulated as the *r*_c_. The *r*_c_ of the three systems of study are summarized in Table 1.

Once the *r*_c_ are determined, the “thiol–epoxy” ratios of study have been experimentally determined by trial and error as follows: starting from *r* = *r*_c_ and increasing by 0.05, bent-shaped samples were prepared and a flat temporary-shape was programmed following the shape-memory programming procedure explained in the next section. In some systems, samples with ratios too close to *r*_c_ could not allow such level of deformation during the programming process and broke. The first ratio in which the sample did not break was stipulated as the initial ratio. From this ratio, increasing by 0.1 each time, the other formulations were defined until *r* ≤ 0.9. Therefore, the selected formulations of study are (S3-DGEBA-*r* = 0.65/0.75/0.85), (S4-DGEBA-*r* = 0.6/0.7/0.8) and (S3-SU8-DGEBA-*r* = 0.6/0.7/0.8). As can be seen, the role of the epoxy excess in the deformation level is the crucial parameter in these systems. Regardless of *r*_c_, in both S4-DGEBA and S3-SU8-DGEBA, working at *r* < 0.6 leads to very brittle materials for such deformation level.

The formulations without SU8 were prepared by pouring the compounds in a glass vial and manually stirring until a homogeneous liquid was attained. In the case of the formulation containing SU8, the DGEBA and SU8 resins were previously mixed by dissolving them in dichloromethane to ensure a homogeneous and compatible mixture. The solvent was then released under vacuum, stirring and temperature. Afterwards, the mixture was cooled down to room temperature and the S3 and 1MI reagents were poured in and the whole mixture manually stirred.

### 2.2. Sample Processing

**Figure 1:**
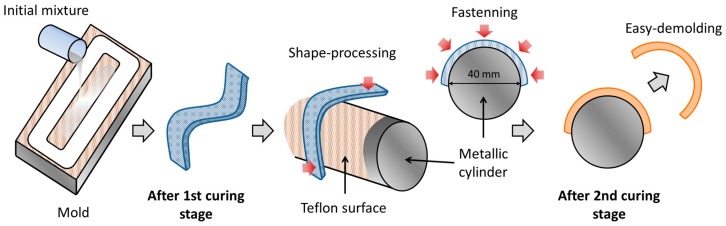
Illustration of the bent-shaped sample processing: from the initial liquid mixture to the final solid bent-shaped material.

Taking advantage of the sequential dual-curing system, it is possible to obtain complex-shaped materials in an easy, efficient and most important, reproducible manner. In this study, bent-shaped SMPs have been processed in order to analyze the SMR in flexural mode; from a flat temporary-shape to the original bent-shape, thus, recreating one of the most usual working designs of an actuator. The entire process to obtain the bent-shaped samples is depicted in Figure 1. First, the initial mixture is prepared as described in the previous section. Once the mixture is prepared, it is rapidly poured into a mold to obtain an intermediate prismatic-shaped material with specific dimensions. The first curing stage is carried out at 50 °C during 3 h to ensure the completion of the “thiol–epoxy” polycondensation [20]. Afterwards, thanks to the high deformability of the intermediate material, it is easily molded onto a metallic cylinder of 40 mm diameter covered with a Teflon layer to obtain the desired level of curvature in the sample. The Teflon layer avoids the sample adhesion onto the metallic surface after the second curing stage. Then, in order to impede the recovery of the original shape due to the viscoelastic response during the temperature increase for the second curing stage, the sample is fastened with minimal force (using sticky Teflon film) onto the cylinder surface. Finally, the second curing stage is carried out at 120 °C for 1 h followed by 1 h at 150 °C to ensure the completion of the epoxy homopolymerization [20].

**Figure 2:**
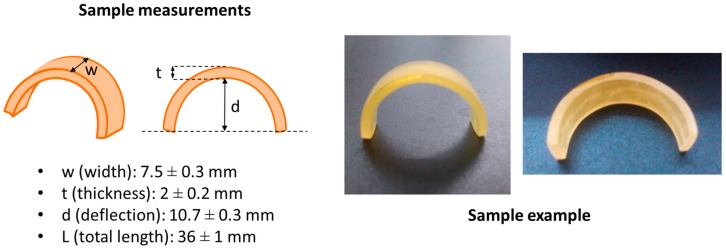
Bent-shaped sample measuring definitions and a photograph of the final sample.

Once the sample is prepared, it is polished in order to eliminate any defects in the surface and to fit the specific dimensions, thus ensuring reproducibility and making possible a safe and meaningful comparison between samples from different formulations and experimental conditions. In Figure 2, a scheme of the sample final dimensions and a photograph of the bent-shaped samples are shown. The deflection, “*d*”, is determined from the lower side of the sample and the other dimensional parameters as schematized.

### 2.3. Thermomechanical Characterization

The S3-SU8-DGEBA system was characterized using a Differential Scanning Calorimeter (DSC) Mettler 821e, calibrated with indium standards. A preliminary characterization of the curing process of S3-SU8-DGEBA formulations was carried at 10 °C/min. Materials with different “thiol–epoxy” ratios ranging from 0 to 1 (0/0.25/0.5/0.75/1) were prepared and analyzed following the same procedure as for the S3-DGEBA and S4-DGEBA systems in our previous work [19]. The intermediate materials were cured in a conventional oven at 50 °C during 3 h. Then, a piece of around 5 mg was analyzed in the DSC from −50 °C up to 100 °C at 10 °C/min. For the final materials, after the 3 h of curing at 50 °C in the oven, a post-curing of 1 h at 120 °C followed by 1 h at 150 °C was imposed and then a piece of around 5 mg was analyzed in the DSC from 30 °C up to 200 °C at 10 °C/min. The glass transition temperatures of the intermediate and final materials were determined as the halfway point in the heat capacity step taking place during the glass transition.

Dynamic mechanical analysis of the formulations of study (S3-DGEBA-*r* = 0.65/0.75/0.85), (S4-DGEBA-*r* = 0.6/0.7/0.8) and (S3-SU8-DGEBA-*r* = 0.6/0.7/0.8) were performed using a DMA Q800 TA Instruments, equipped with a 3-point-bending clamp (15 mm), in oscillation mode at 1 Hz and 0.1% of amplitude strain and imposing a temperature ramp of 3 °C/min. The glassy (*E*_g_) and rubbery (*E*_r_) moduli were determined at 40 °C and *T*_g_ + 30 °C, respectively. The *T*_g_ was determined as the tanδ peak temperature. The height of the peak and width at half-height (FWHM) of the tanδ curve were also determined. The relation between *E*_g_ and *E*_r_ was calculated as a measure of the SMR (efficiency fixing the temporary-shape and recovering the original shape).

### 2.4. Shape-Memory Characterization

**Figure 3:**
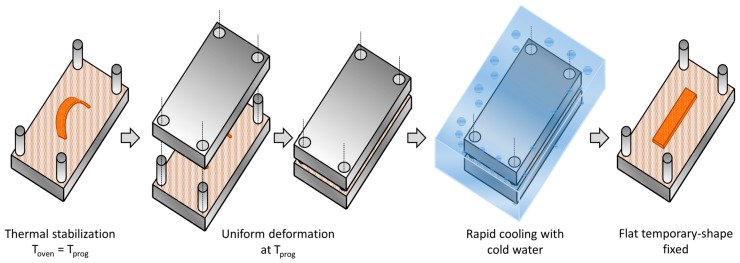
Illustration of the shape-memory programming process.

The shape-memory characterization was divided in two steps: programming of the temporary-shape and recovery of the original shape. For the programming of the temporary-shape, a tailor-made device consisting of male and female stainless steel plates of 15 × 8 cm^2^ of surface was used. In order to produce an equal, homogeneous and controlled pressure over the sample during the programming, the thickness of the female part was fitted to weigh 0.5 kg. The programming procedure is schematized in Figure 3: the male part of the device with the bent sample placed on it, and the female part were introduced separately in a preheated oven at the programming temperature *T*_prog_ = *T*_g_ + 30 °C and held during 30 min for thermal stabilization in order to ensure that the network structure of the material is fully relaxed before programming. Afterwards, the female part of the device was fitted to the male part, and its weight produced the flattening of the sample. The whole assembly was placed immediately inside a bath with cold water for 1 min to ensure that the sample temperature decreased below the network relaxation temperature and therefore fix the temporary-shape. Finally, the female part was removed and the flat sample was wiped dry using a paper towel.

**Figure 4:**
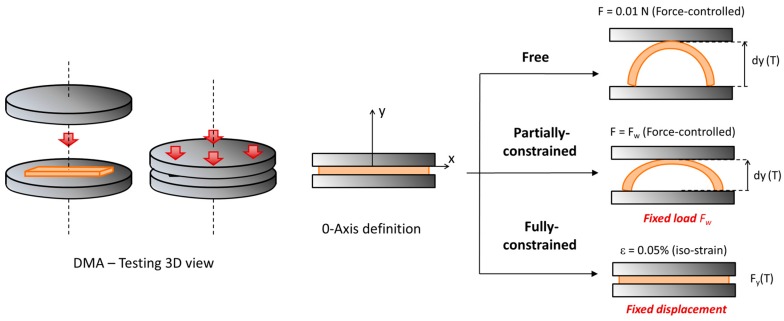
Illustration of the DMA testing procedure including the different recovery scenarios.

Once the samples were programmed, the recovery of the original shape was carried out using the DMA equipped with the compression clamp (diameter of 40 mm) at different operational modes depending on the experiment performed: unconstrained, partially-constrained or fully-constrained recovery conditions. The sample mounting and the recovery analysis is depicted in Figure 4: in unconstrained-recovery experiments, the DMA was set in Force-controlled mode and a minimal force of 0.01 N was imposed to register the recovery-process. The displacement of the moveable clamp, *d*_y_(*T*), was recorded as a measure of the shape-recovery (*SR*), which was determined applying Equation (1). It should be note that in Equation (1) the maximum displacement is defined by the deflection, “*d*”, of the original shape.

The instantaneous shape-recovery speed, *SR*_speed_(*T*), was calculated using Equation (2) as a measure of the shape-recovery sharpness. Using the *SR*_speed_(*T*) curve, the temperature at which the maximum recovery speed is found was determined as the peak of the curve (*T*_peak_), and the duration of the process as the width of the curve at half-height (∆*T*_peak_). Additionally, common shape-memory quantifiers, such as, the shape-recovery rate (*V*_r_) and the shape-recovery ratio (*R*_r_) were determined for comparison purposes among the different formulations. *V*_r_ was determined from *SR*(*T*_1_) = 0.15 to *SR*(*T*_2_) = 0.85 applying Equation (3) and *R*_r_ applying Equation (4), where *d*_y_(*T*_end_) is the displacement reached by the moveable clamp at the end of the recovery-process.

(1) $$SR\left(T\right)=1-\frac{d-d_{y}\left(T\right)}{d}$$

(2) $$SR_{\mathrm{speed}}\left(T\right)=\frac{\partial SR\left(T\right)}{dT}$$

(3) $$V_{r}=\frac{SR_{15\%-85\%}}{∆T_{{\mathrm{SR}}_{15\%-85\%}}}\cdot \frac{dT}{dt}$$

(4) $$R_{r}=\frac{d_{y}\left(T_{\mathrm{end}}\right)}{d}\cdot 100$$

The partially-constrained experiments were carried out in Force-controlled mode applying a constant force (*F*_w_) equal in all the experiments. The displacement of the moveable clamp was recorded and the shape-recovery was measured using Equation (1). Applying Equation (2), the *SR*_speed_(*T*) curve was calculated and both *T*_peak_ and ∆*T*_peak_ were determined as explained above. Additionally, the relative work, *W*_rel_, developed by the sample was determined both, experimentally and theoretically, using Equation (5) (in mN·mm/mm). In this expression, the final displacement reached, *d*_y_(*T*_end_), has been normalized with respect to the maximum displacement, “*d*”, for comparison purposes.

(5) $$W_{\mathrm{rel}}=F_{w}\cdot \frac{d_{y}\left(T_{\mathrm{end}}\right)}{d}$$

For the calculus of the theoretical relative work, *W*_rel_^t^, it has been taken into account that all the force given by the sample during the SME is equal to the force given to program the sample into the temporary-shape (*F*_prog_). *F*_prog_ can be approached using the beam deflection formula for small deformations given by Equation (6).

(6) $$F_{\mathrm{prog}}=\frac{4\cdot \left(d+\frac{t}{2}\right)\cdot E_{\left(T_{\mathrm{prog}}\right)}\cdot w\cdot t^{3}}{L^{3}}$$

where “*d*” is the deflection of the sample, *E*_(*T*_prog_)_ is the modulus at the programming temperature, considered constant and equal to the relaxed modulus *E*_r_, and “*w*”, ”*t*” and “*L*” are the dimensions of the sample (width, thickness and length, respectively). Once *F*_prog_ is determined, the theoretical displacement reached, *d*_y_(*T*_end_), can be determined reordering Equation (6) into (7) and taking into account the force applied by the DMA, *F*_w_, behaving in opposite direction during the recovery experiment. Then, by simply applying Equation (5), *W*_rel_^t^ is determined.

(7) $$d_{y}\left(T_{\mathrm{end}}\right)=\frac{\left(F_{\mathrm{prog}}-F_{w}\right)}{4\cdot L^{3}\cdot E_{\left(T_{\mathrm{prog}}\right)}\cdot w\cdot t^{3}}-\frac{t}{2}$$

The fully-constrained experiments were performed at Iso-Strain mode imposing 0.05% of strain in order to measure the force generated by the sample, *F*_y_(*T*), during the recovery-process. In the same way as for the unconstrained and partially-constrained experiments, *T*_peak_ and ∆*T*_peak_ were determined from the derivative curve, *dF*_y_(*T*)/*d*_T_. As proposed by Li and Wang [26], *F*_y_(*T*) can be divided in different components as shown in Equation (8). *F*_residual_(*T*) is the force generated due to incomplete stress relaxation during the programming process. In our case, it can be neglected since the programming process is performed at *T*_prog_ >> *T*_g_, thus, the sample is able to relax all the structural changes during the programming. *F*_thermal_(*T*) is the force generated due to the thermal expansion of the sample and it depends on the contact area of the sample with the DMA clamp. *F*_SR_(*T*) is the force generated due to the SME during the mechanical relaxation and it depends on the programming and recovery time-temperature conditions.

(8) $$F_{y}\left(T\right)=F_{\mathrm{SR}}\left(T\right)+F_{\mathrm{residual}}\left(T\right)+F_{\mathrm{thermal}}\left(T\right)$$

The thermal force, *F*_thermal_(*T*), has a crucial role and, in contrast with common experiments carried out in tension mode, it acts in the same direction of the SME. It can be approached by applying Equation (9), which takes into account the variation of the modulus with the temperature.

(9) $$F_{\mathrm{thermal}}=\;E\left(T\right)\cdot \alpha \left(T\right)\cdot \left(T-T_{0}\right)\cdot S_{\mathrm{eff}}$$

where *E*(*T*) is the modulus at temperature “*T*”, *α*(*T*) is the thermal expansion coefficient which can be considered a constant value in the ranges *T* < *T*_g_ and *T* > *T*_g_ (4.5 × 10^−5^ and 6.0 × 10^−6^ °C^−1^ respectively, values obtained from common epoxy materials in the literature [27]). *T*_0_ is the initial temperature (*T*_0_ = 30 °C in all the experiments) and “*S*_eff_” is the effective section (section in contact with the DMA clamp). Theoretically, in a compression experiment “*S*_eff_” would be the whole contact surface (width *x* length) of the sample; however, in spite of the good fixation of the temporary-shape, some residual shape-recovery takes place, therefore the effective surface approaches a punctual contact instead. To better calculate “*S*_eff_”, one can take advantage of the force given by the DMA to keep the imposed ε of 0.05%. This force is applied at room temperature and therefore, by simply applying Hooke’s law it is possible to obtain the real surface in which it is being applied (see Equation (10)).

(10) $$S_{\mathrm{eff}}=\;\frac{F_{\mathrm{DMA}}}{E_{g}\cdot \varepsilon }$$

where *E*_g_ is the modulus in the glassy state (at *T*_0_ = 30 °C) and *ε* is the imposed deformation in the experiment (0.05%).

Finally, the force generated due to the SME during the mechanical relaxation, *F*_SR_(*T*), has been determined by subtracting *F*_thermal_(*T*) from *F*_y_(*T*). From this curve, the total shape-recovery force F_SR_ has been determined as the force gained between the onset and offset points. Note that the force generated in fully-constrained experiments shows the typical behavior of the network relaxation processes, thus, an onset and offset points can be defined.

## 3. Results and Discussion

### 3.1. DSC Analysis of the Dual-Curing Process

**Figure 5:**
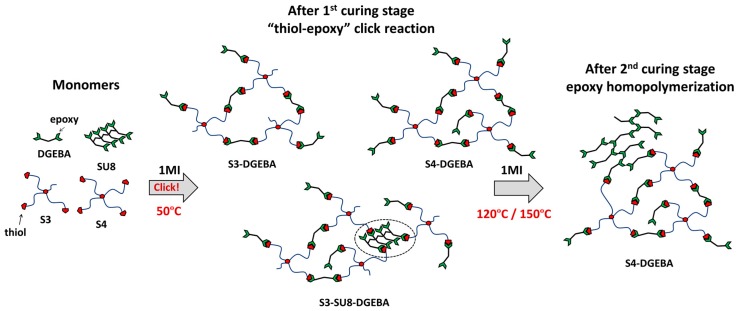
Monomers and expected network structures after the first and second curing stages for all the systems of study.

As it was explained in our previous works [19,20], sequential dual-curing systems are attained by preparing different “thiol–epoxy” mixtures in off-stoichiometric proportions. The use of tertiary amines as catalyst allows the separation of both polymerization processes, the initial polycondensation between thiol and epoxy groups and the following homopolymerization of the remaining unreacted epoxy groups. The monomers and expected networks formed after the “thiol–epoxy” polycondensation (1st curing stage) and epoxy homopolymerization (2nd curing stage) are shown in Figure 5. As it can be seen, the “thiol–epoxy” click polycondensation takes places rapidly at low temperature, leading to partially-crosslinked network structures. These intermediate materials are stable at room temperature until the temperature is increased and the remaining epoxy groups react forming a highly crosslinked and less flexible network structure, enhancing the thermomechanical properties of the material.

**Figure 6:**
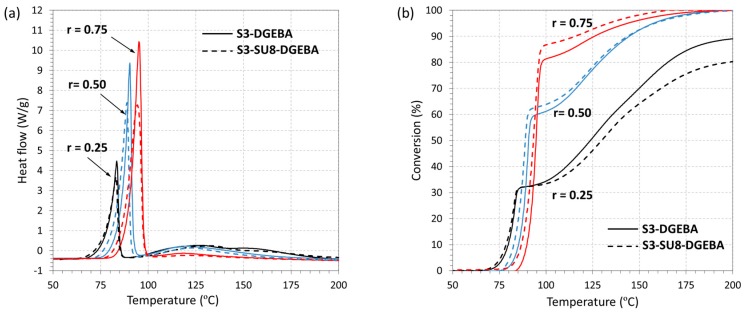
DSC traces of the curing process of the S3-SU8-DGEBA and S3-DGEBA systems at different “thiol–epoxy” ratios: (**a**) heat flow trace; and (**b**) conversion trace.

In order to analyze the duality of the new system, the curing process of both S3-SU8-DGEBA and S3-DGEBA systems at different “thiol–epoxy” ratios are illustrated in Figure 6. In Figure 6a, the heat flow is plotted as a function of the temperature. The presence of two well-separated peaks in is an indication of two sequential polymerization processes taking place, in agreement with our previous results [20]. The first and sharper peak is related to the “click” thiol–epoxy polycondensation, while the broader and smaller peak to the anionic epoxy homopolymerization of the excess epoxy groups. Therefore, the incorporation of SU8 does not affect the duality of the curing process. In Figure 6b the conversion is plotted as a function of the temperature. Note that the conversion curves (Figure 6b) have been determined taking into account empirical values of the enthalpy of reaction from the literature: the thiol–epoxy polycondensation (1st curing process) releases around 130 kJ/eq., while the epoxy homopolymerization around 100 kJ/eq. [28,29]. Within experimental error, the contribution of the first process to the total conversion is proportional to the presence of thiol in the mixture and is not affected by the presence of SU8, as could be expected.

In Table 2, the enthalpy values corresponding to each polymerization process are presented. As can be seen, the “thiol–epoxy” polycondensation (1st curing process) releases around 130 kJ/eq., similar values to the reported one. Nevertheless, during the epoxy homopolymerization, formulations containing high content of epoxy excess (*r* = 0.25), do not reach the expected enthalpy value. Moreover, this is more evident in the S3-SU8-DGEBA system; the enthalpy per equivalent epoxy is 79.1 kJ/eq., while in the S3-DGEBA system is 84.4 kJ/eq., both of them lower than 100 kJ/eq. This may be caused by topological restrictions in the highly crosslinked homopolymer network, especially in the presence of SU8, leading to incomplete reaction of epoxy groups.

### 3.2. Thermomechanical Results

**Figure 7:**
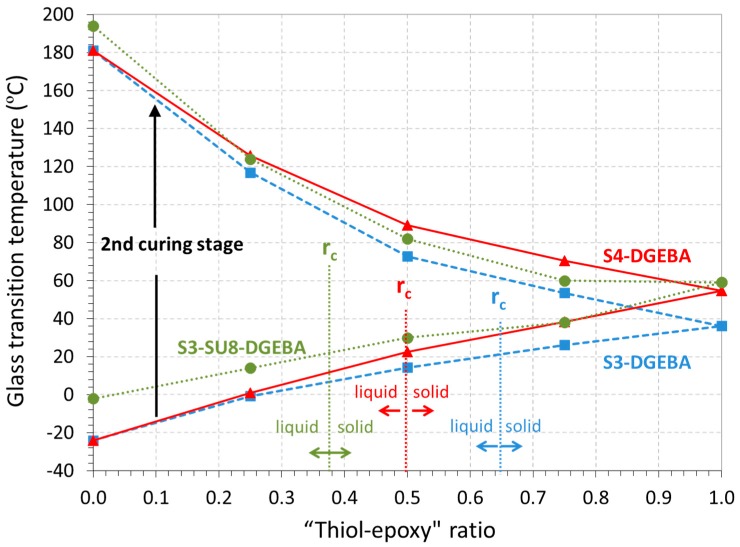
Glass transition temperatures and critical ratio for the different systems of study at different “Thiol–epoxy” ratios.

In Figure 7, the glass transition temperatures (*T*_g_) of the intermediate materials (lower curves) and final materials (upper curves) are shown for all the systems of study at the different “thiol–epoxy” ratios (the data for S3-DGEBA and S4-DGEBA are taken from our previous work [19]). Overall, two different trends are shown for each material: the *T*_g_ of the intermediate materials increase with increasing “thiol–epoxy” ratio due to the decreasing amount of unreacted excess DGEBA at the end of the first stage of the curing process (see schemes in Figure 5). In contrast, the *T*_g_ of the final materials increases with decreasing “thiol–epoxy” ratio because of the higher contribution of the homopolymerization of the excess epoxy groups. The increase of functionality of the crosslinking agents, S3 and S4, results in somewhat different properties at high “thiol–epoxy” ratios and reduces the critical ratio, *r*_c_. In contrast, the incorporation of the high functional resin SU8 also produces an increase in *T*_g_ at lower “thiol–epoxy” ratios and also reduces significantly the critical ratio *r*_c_ because of its higher functionality. The *T*_g_ of the uncured neat epoxy formulation (without thiol) increases from −23 to −1 °C when SU8 is added to the formulation, due to the lower mobility of the highly functional SU8 monomer. The final *T*_g_ of the neat epoxy homopolymer increases from 181 to 193 °C when SU8 is added. This small increase in the final *T*_g_ of the homopolymer can be explained by the fact that conversion of epoxy groups may not be complete due to topological restrictions, as explained in the preceding section. Overall, the possibility to modulate the structural properties of the materials and the critical ratio of the systems by combining different thiol and epoxy compound makes it possible to extend the processing of complex-shaped materials to lower “thiol–epoxy” ratios and therefore makes it possible to get materials with also different structural properties.

Table 3 shows the results of the thermomechanical characterization of the cured materials using DMA. In agreement with the above explanation, on decreasing the “thiol–epoxy” ratio, i.e., increasing the amount of epoxy homopolymerization, the *T*_g_ of the materials increases significantly. In the same way, the relaxed modulus, *E*_r_, increases and the relaxation process becomes more heterogeneous (FWHM increases and the peak of the tanδ curve decreases). It is well known that the increase of the crosslinking density leads to higher relaxed modulus and a more heterogeneous network structure with longer relaxation times. The incorporation of a highly functional and rigid epoxy resin (SU8) to the S3-DGEBA system has an evident impact on the structural properties, showing a noticeable increase in *E*_r_ and *T*_g_ and a significant broadening of the network relaxation process, as deduced from the increase in FWHM (i.e., from 12.1 °C in the S3-DGEBA-0.75 to 22.8 °C in the S3-SU8-DGEBA-0.7) and the decrease in the tanδ peak height.

In terms of shape-memory properties, the relation *E*_g_/*E*_r_ is higher of 100 in all cases; this means two orders of magnitude, which is sufficiently high to expect good SMR in terms of shape-fixation and -recovery. This parameter increases with the decrease of *E*_r_ on approaching the stoichiometric ratio because, in all these materials, the value of the glassy modulus *E*_g_ is similar regardless of the structural differences and therefore the relation is governed by *E*_r_. On the other hand, the homogeneity of the relaxation process (quantified by the breadth of tanδ) gives us an idea of the shape-recovery rate [21]. The S3-SU8-DGEBA materials have broader relaxation processes and therefore it is expected a slower and more gradual recovery-process in comparison with the other systems.

### 3.3. Shape-Memory Results

**Figure 8:**
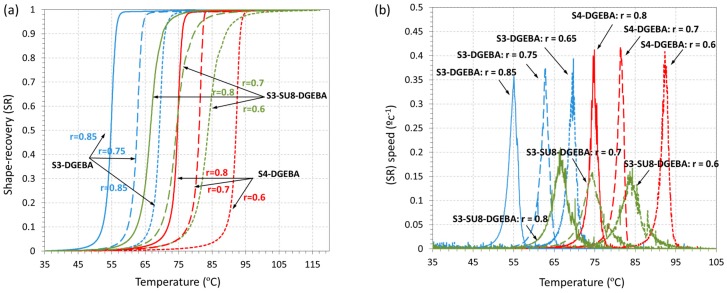
Shape-memory unconstrained recovery experiments: (**a**) shape-recovery as function of temperature; and (**b**) derivative of shape-recovery with respect to temperature (*SR*_speed_) as function of temperature.

As stated in the experimental part, the shape-memory characterization has been studied under three different scenarios (see Figure 4). The results of the unconstrained recovery experiments are shown in Figure 8 and the parameters of interest summarized in Table 4. First, it should be pointed out that all the samples were able to completely recover the original shape, with values of *R*_r_ almost equal to 100% in all cases. This can be explained by the safe programming conditions and the structural properties of epoxy-based SMPs [16]. The programming was carried out at *T*_prog_ >> *T*_g_ and under mild deformation conditions, therefore completely elastic deformation was imposed, where no damaging processes and no stress relaxation were involved.

The evolutions of the shape-recovery (*SR*) and recovery speed (*SR*_speed_) parameters show a clear difference between the formulations without and with SU8. The shape-recovery process is considerably faster and sharper in the materials without SU8, regardless of the use of S3 or S4 as crosslinking agent. The *SR*_speed_ curves of S4-DGEBA formulations are slightly taller and narrower than those of S3-DGEBA formulations, but, if one analyzes the overall recovery rate *V*_r_ (see Table 4), it can be seen that they have very similar and high values (70%–80%/min). In contrast, the presence of SU8 leads to lower and broader *SR*_speed_ curves (∆*T*_peak_ increases from 2 °C up to 4–5 °C), and therefore lower *V*_r_ values (around 35%/min). Moreover, the effect of the “thiol–epoxy” ratio is more relevant with the presence of SU8, leading to a clear decrease in *V*_r_ from 46% to 35% on increasing the epoxy content from *r* = 0.8 to *r* = 0.6 in the S3-SU8-DGEBA system, while this is not that evident in the S3-DGEBA and S4-DGEBA systems. This must be caused by the effect of the strongly hindered homopolymer structure of SU8 in comparison with DGEBA.

Parameters as ∆*T*_peak_ and *T*_peak_ are closely related to the glass transition. *T*_peak_ is commonly close to the *T*_g_ nominal value and ∆*T*_peak_ is related to the breath of the tanδ curve. As it can be seen, in all the experiments, *T*_peak_ is placed slightly below *T*_g_^DMA^: however it increases with the increase in epoxy content accordingly to the trend observed in the *T*_g_^DMA^. Moreover, the differences regarding ∆*T*_peak_ are clearly related to the structural relaxation. As explained above, formulations containing SU8 show higher ∆*T*_peak_ due to the broader relaxation process as deduced from the FWHM values in Table 2. These results point out the close relation between network relaxation and shape-recovery in glassy materials, as previously observed [22].

**Figure 9:**
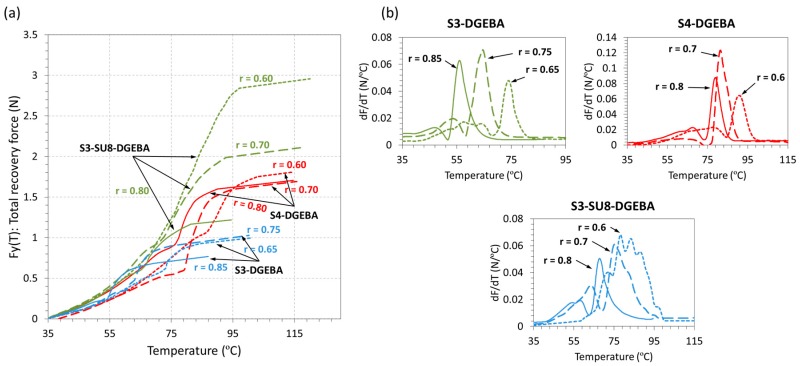
Shape-memory fully-constrained recovery experiments: (**a**) force generated as a function of the temperature; and (**b**) derivative of the force generated with respect to the temperature as a function of the temperature.

Fully-constrained experiments were carried out to determine the response of the material under a completely impeded scenario. The DMA was set in Iso-Strain mode and the force, *F*_y_(*T*), generated by the sample during the recovery-process was recorded. The results are shown in Figure 9 and the parameters of interest summarized in Table 4. In a similar way to the *SR*_speed_ (Figure 8b), the force rate, *dF*_y_/*d*_T_, in (N/°C) has been determined and presented as small side graphics on the right side of the figure for all the formulations under study.

The recovery-process can be divided into three different sections (see Figure 9a). First, there is a constant increase of the force mostly related to the thermal expansion of the material at temperatures below *T*_g_. Then, on approaching to *T*_g_, the material undergoes an initial relaxation process caused by the lowering of the modulus followed by a drastic increase of the force due to the SME. This is clearly appreciated in the N/°C graphics on Figure 9b, showing an initial and small decreasing peak followed by a drastic increasing peak. Finally, the force slightly increases probably due to the thermal expansion at temperatures above *T*_g_. The effect of the network structure is clearly appreciated in the homogeneity of the recovery-process, as deduced from the shape of the, *dF*_y_/*d*_T_, curves (Figure 9b). Formulations containing SU8 show broader and slower processes, while S3-DGEBA and S4-DGEBA systems have sharper and faster processes. Moreover, the effect of the presence of the SU8 molecule is clearly appreciated; on decreasing the ratio to 0.6 in the S3-SU8-DGEBA system, up to three peaks appear during the SME, pointing out the complex relaxation process produced by the presence of SU8 in the thiol–epoxy and epoxy homopolymer networks.

On analyzing the parameters obtained from *dF*_y_/*d*_T_, it can be seen that both, ∆*T*_peak_ and *T*_peak_, are clearly related to the glass transition process and network structural properties, in accordance with the unconstrained experiments. Nevertheless, the magnitude of ∆*T*_peak_ is considerably higher in all the formulations of study. Moreover, the differences regarding the content of SU8 are more evident in the S3-SU8-DGEBA system, ∆*T*_peak_ increases from 7 °C at *r* = 0.8 to 23.9 °C at *r* = 0.6. This suggests that in completely impeded scenarios, differences regarding the crosslinking points are more relevant than in unconstrained experiments [30], and that, overall, the recovery-process is extended longer.

**Figure 10:**
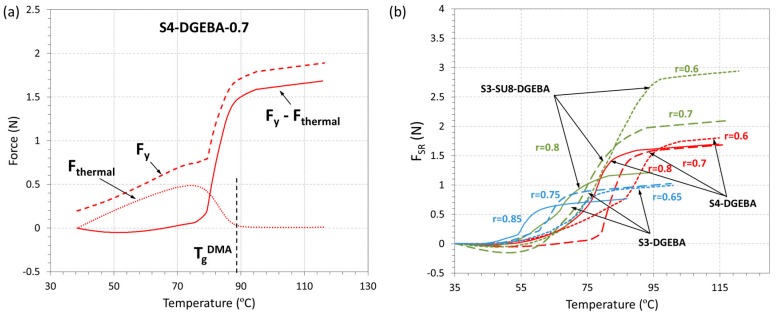
Shape-memory fully-constrained recovery experiments: (**a**) thermal force subtraction process; (**b**) force generated after subtracting the thermal contribution.

The thermal force, *F*_thermal_(*T*), and its effect on *F*_y_(*T*) is shown in Figure 10. In Figure 10a, *F*_thermal_(*T*) is plotted as a function of the temperature and subtracted from *F*_y_(*T*) to obtain the shape-recovery force, *F*_SR_(*T*), for the formulation S4-DGEBA-0.7, while, in Figure 10b, the *F*_SR_(*T*) curves are presented for all the formulations studied. As can be seen in Figure 10a, the higher contribution of *F*_thermal_(*T*) takes place at *T* < *T*_g_; *F*_thermal_(*T*) increases until a maximum is reached, and then goes down to a minimum and almost constant value. This is caused by the drastic decrease of the modulus as the material becomes a rubber. Having in mind these results, the resulting curves *F*_SR_(*T*) = *F*_y_(*T*) − *F*_thermal_ (Figure 10b) clearly point out that, as stated above, the thermal expansion is the responsible of the initial increase of *F*_y_(*T*), while *F*_SR_(*T*) is mainly related to the network relaxation and build-up of stress in the fully-impeded recovery process taking place during heating. This result emphasizes the relevance of the thermal expansion in fully-constrained experiments at temperatures below *T*_g_ and should be taken into account in certain applications to avoid an early undesired actuation.

The value and evolution of F_SR_ is closely related to the network structural properties. In general, formulations with higher relaxed modulus (see Table 2) lead to higher *F*_SR_ values (SU8 > S4 > S3 see in Table 4). However, there is a clear difference in the presence of SU8, which may be connected to its molecular structure. Moreover, on decreasing the ratio in the S3-SU8-DGEBA system, i.e., increasing the epoxy homopolymer content, *F*_SR_ increases much more than in the other cases.

**Figure 11:**
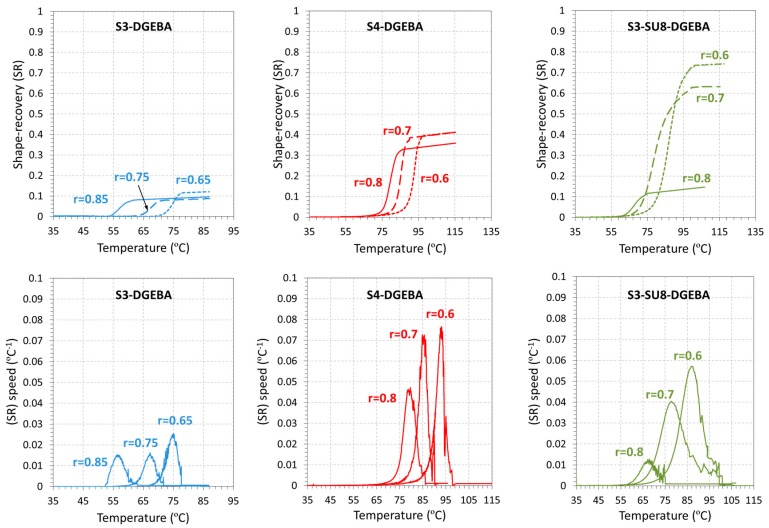
Shape-memory partially-constrained recovery experiments: shape-recovery as a function of the temperature.

Partially-constrained experiments were performed to fully characterize the SMR in more realistic applications since most of the SMPs are required to recover their shape working against a force. A constant force, *F*_w_, is applied to the sample and the work done by the sample during the recovery-process is calculated. *F*_w_ is fixed to a constant value for all the experiments. As can be seen in Table 4, the minimum force to ensure that all the formulations are able to produce a positive work is 0.5 N (the S3-DGEBA-0.85 gives the minimum *F*_SR_ value of 0.53 N). The shape-recovery curves (*SR*) and the shape-recovery instantaneous speed *SR*_speed_ are shown in Figure 11, and the parameters of interest summarized in Table 4.

First, it must be noted that all the samples were able to partially recover the original shape, that is, a positive work was produced. The shape of the (*SR*) curves is similar to that of the unconstrained recovery experiments, but the *SR*_speed_ curves are broader, as in fully-constrained experiments. In contrast, the influence of the crosslinking points is not evident in partially-constrained experiments. ∆*T*_peak_ is similar or even lower in formulations with higher content of epoxy (i.e., ∆*T*_peak_ of S3-SU8-DGEBA-0.7 is 12.1, while it goes down to 10.9 in S3-SU8-DGEBA-0.6). In general, *T*_peak_ is closely related to *T*_g_^DMA^ in all experimental modes, while ∆*T*_peak_ increases from unconstrained to fully-constrained modes, pointing out an extension of the recovery-process caused by the presence of an external force impeding or making difficult the recovery of the original shape.

**Figure 12:**
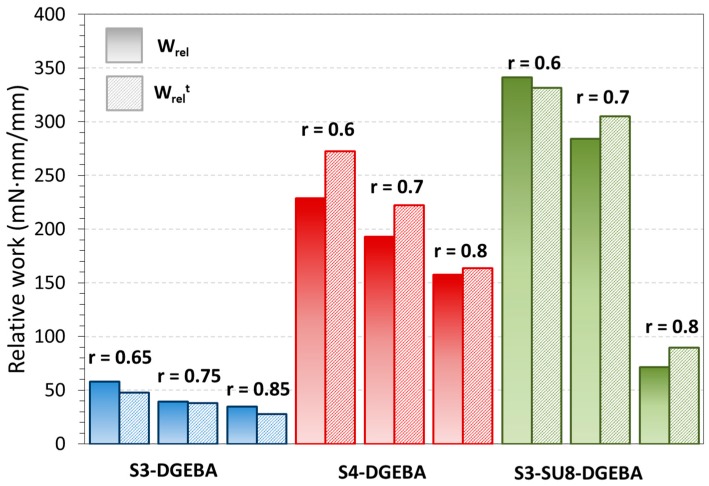
Comparison between predicted *W*_rel_^t^ and experimental *W*_rel_ for all the formulations of study.

The experimental relative work and the theoretical relative work, *W*_rel_, are shown in Figure 12. As mentioned in the experimental section, the work has been normalized to a relative work (mN·mm/mm) in order to allow comparison between samples taking into account the differences in the deflection. The increase in functionality of the crosslinker compound (SU8 > S4 > S3), as well as the presence of the SU8 in the network structure clearly enhance the mechanical performance of the actuator, in line with the results from the fully-constrained experiments. *W*_rel_ increases on increasing *F*_SR_, because the sample is able to produce a higher displacement of the clamp during the SME. Moreover, it is clearly appreciated an increasing trend of *W*_rel_ on decreasing the “thiol–epoxy” ratio in all the systems of study. These results point out that the increase of the crosslinker functionality (the presence of the DGEBA homopolymer) has a positive effect in the force generated during the recovery-process as shown in the fully-constrained experiments.

As stated in the experimental part, it is possible to predict the theoretical relative work, *W*_rel_^t^ as follows: the theoretical *d*_y_(*T*_end_) reached by the sample during the recovery-process is approached by using Equations (6) and (7) and then *W*_relt_ is determined by simply applying Equation (5) with the predicted *d*_y_(*T*_end_). Through this methodology, one considers that the sample produces a *F*_SR_ equal to *F*_prog_ as no energy losses takes place during the programming and thus, all the programmed force is then released during the recovery-process. In Figure 12, the comparison between the experimental relative work generated and the predicted one is presented.

As it can be observed, the use of the beam formula for small deformations gives reliable and good results; the predicted values fit very well with the experimental ones. This can be explained as follows: on one side, during the programming process, the sample is above *T*_g_ and the network structure completely relaxed, therefore, the response of the sample to the applied stress is completely elastic. On the other side, the recovery-process takes place in between and above *T*_g_, with a constant force applied, thus, the time-scale of the experiment is high enough to allow almost complete relaxation of the network during the process. This means that the sample response is almost completely elastic to the stress applied in both, the programming and the recovery processes; therefore, the equation is able to predict properly the final deflection and/or force applied.

## 4. Conclusions

Bent-shaped shape-memory actuators have been successfully characterized by taking advantage of the sequential dual-curing of off-stoichiometric “thiol–epoxy” mixtures. By appropriately choosing the “thiol–epoxy” ratio, solid-like and conformable materials are formed after the first curing stage that can be easily processed into bent-shaped materials and fixed thanks to the crosslinks formed in the second curing stage. The SMPs have been fully characterized as potential actuators in different shape-memory scenarios: unconstrained, partially-constrained and fully-constrained. The unconstrained recovery experiments showed an efficient and tailorable recovery process; the SMPs are able to efficiently fix the temporary-shape and completely recover their original shape with high recovery rates. The dependence of the recovery-process on the network structure, in particular on the network relaxation process, has been clearly evidenced in the presence of the high functional epoxy resin SU8 in the S3-DGEBA system, leading to more heterogeneous network structures and thus lower recovery rates.

The fully-constrained experiments showed the capability of these materials to release a force instead of recovering the original shape on heating above *T*_g_. This force has been found closely related to the network structure and chemical structure of the crosslinking points. The presence of crosslinks of higher functionality increase the force released during the SME. Moreover, large and spatially impeded crosslinks (SU8 molecule) produce a major effect on the force enhancement.

In partially-constrained experiments, the materials showed the capability to produce a positive work on heating above *T*_g_. The work released shows an increasing trend with the increase of the crosslinks functionality and size (SU8 > S4 > S3), as well as with the increase of epoxy excess accordingly to the force released in the fully-constrained experiments. Moreover, it has been shown that it is possible to efficiently predict the work released by applying the beam deflection formula for small flexural deformations only taking into account the relaxed modulus and dimensions of the sample.

In general, the recovery-process is closely related to the network structure and its relaxation process in all experimental setup. The temperature at which the maximum rate is found (*T*_peak_) is closely related to the *T*_g_ nominal value, while the heterogeneity of the relaxation process is connected with the length or duration of the recovery-process, which depends on the experimental setup and increases according to the constraint level, from unconstrained to fully-constrained experiments.

## Figures and Tables

**Table 1:** Composition of the different formulations of study.

System	Critical ratio (*r*_c_) ^1^	“Thiol–Epoxy” ratio	Thiol (wt %)	DGEBA (wt %)	SU8 (wt %)	1MI (wt %)
S3-DGEBA	0.65	0.65	31.86	67.15	-	0.99
		0.75	35.03	63.98	-	0.99
		0.85	37.91	61.10	-	0.99
S4-DGEBA	0.5	0.60	28.43	70.58	-	0.99
		0.70	31.65	67.36	-	0.99
		0.80	34.59	64.42	-	0.99
S3-SU8-DGEBA	0.38	0.60	29.14	46.58	23.58	0.99
		0.70	32.42	44.42	22.49	0.99
		0.80	35.42	42.45	21.49	0.99

^1^ Experimentally determined from rheological analysis.

**Table 2:** Reaction heat of the first and second polymerization processes corresponding to the curing of S3-DGEBA and S3-SU8-DGEBA formulations with a thiol: epoxy ratios of 0.25, 0.50 and 0.75.

Formulation	∆*H*_1st_ (J/g)	∆*H*_2nd_ (J/g)	∆*H*_1st_ (kJ/eq.) ^1^	∆*H*_2nd_ (kJ/eq.) ^1^
S3-DGEBA-0.25	151	291	131.6	84.4
S3-DGEBA-0.50	263	197	132.0	98.7
S3-DGEBA-0.75	348	82	131.9	93.2
S3-SU8-DGEBA-0.25	147	261	133.8	79.1
S3-SU8-DGEBA-0.50	257	190	134.2	99.1
S3-SU8-DGEBA-0.75	347	83	136.3	97.8

^1^ Heat flow per equivalent epoxy (MW_eq-epoxy_ determined as an average value taking into account the DGEBA:SU8 proportion).

**Table 3:** Thermomechanical and structural properties of the different formulations of study.

Formulation	S3-DGEBA			S4-DGEBA			S3-SU8-DGEBA		
“thiol–epoxy” ratio	0.65	0.75	0.85	0.6	0.7	0.8	0.6	0.7	0.8
*E*_g_ (MPa)	2150	2001	2130	2200	2130	2150	2310	2250	2310
*E*_r_ (MPa)	10.9	9.6	8.4	18.4	15.1	14.0	19.5	17.3	14.4
*T*_g_^DMA^ (°C)	80.1	70.0	61.0	98.0	89.0	81.0	96.0	81.0	73.0
FWHM (°C)	12.8	12.1	11.0	11.5	10.5	10.0	23.0	22.8	21.8
tanδ peak	1.25	1.35	1.46	1.16	1.23	1.30	0.69	0.81	0.92
*E*_g_/*E*_r_	197	208	254	120	141	154	118	130	160

**Table 4:** Parameters obtained from the shape-memory analysis (unconstrained, partially-constrained and fully-constrained) of the different formulations studied: Peak temperature (*T***_peak_**), Width at half-height of the peak (**∆***T***_peak_**), Recovery-rate (*R***_r_**), Relative Work output (*W***_rel_**) and shape-recovery force (*F***_SR_**).

	Unconstrained				Partially-constrained			Fully-constrained		
Formulation	*R*_r_ (%)	∆*T*_peak_ (°C)	*T*_peak_ (°C)	*V*_r_ (%/min)	∆*T*_peak_ (°C)	*T*_peak_ (°C)	*W*_rel_ (mN·mm/mm)	∆*T*_peak_ (°C)	*T*_peak_ (°C)	*F*_SR_ (N)
S3-DGEBA-0.65	99.9	2.0	69.7	73.7	4.0	75.0	57	5.0	74.0	0.74
S3-DGEBA-0.75	100.0	2.0	62.9	76.7	4.7	67.2	39	6.2	64.5	0.76
S3-DGEBA-0.85	99.5	2.3	55.0	80.3	5.2	56.7	34	5.4	56.2	0.53
S4-DGEBA-0.60	99.9	1.7	92.3	79.2	3.7	93.2	187	7.6	91.0	1.71
S4-DGEBA-0.70	99.9	1.8	81.3	72.1	4.3	85.7	179	6.7	81.8	1.42
S4-DGEBA-0.80	99.8	1.8	74.9	79.0	6.2	79.6	136	5.6	78.4	1.47
S3-SU8-DGEBA-0.60	99.8	4.9	84.3	34.7	10.9	87.2	314	23.9	81.9	2.92
S3-SU8-DGEBA-0.70	99.8	4.8	74.4	37.7	12.1	77.7	247	12.4	77.8	1.79
S3-SU8-DGEBA-0.80	99.9	4.1	66.5	45.8	7.8	67.9	49	7.0	68.3	0.89
